# Supplementary material for: Clinical validation of engineered CRISPR/Cas12a for rapid SARS-CoV-2 detection
Source: Commun Med (Lond). 2022 Jan 12;2:7. doi: 10.1038/s43856-021-00066-4 (PMC9053293; doi:10.1038/s43856-021-00066-4)
Supplement: Supplementary file 4 — Supplementary Information [file 43856_2021_66_MOESM4_ESM.pdf]

SUPPLEMENTARY MATERIALS

Clinical Validation of Engineered CRISPR/Cas12a For Rapid SARS-CoV-2 Detection

Long T. Nguyen[1], Santosh R. Rananaware[1], Brianna L.M. Pizzano[2], Brandon T. Stone[3], Piyush K. Jain[1,4] *

[1]Department of Chemical Engineering, University of Florida, Gainesville FL, USA

[2]Department of Agricultural and Biological Engineering, University of Florida, Gainesville FL, USA

[3]Department of Microbiology, University of Florida, Gainesville FL, USA

[4]UF Health Cancer Center, University of Florida, Gainesville FL, USA

*Corresponding author: Jainp@ufl.edu

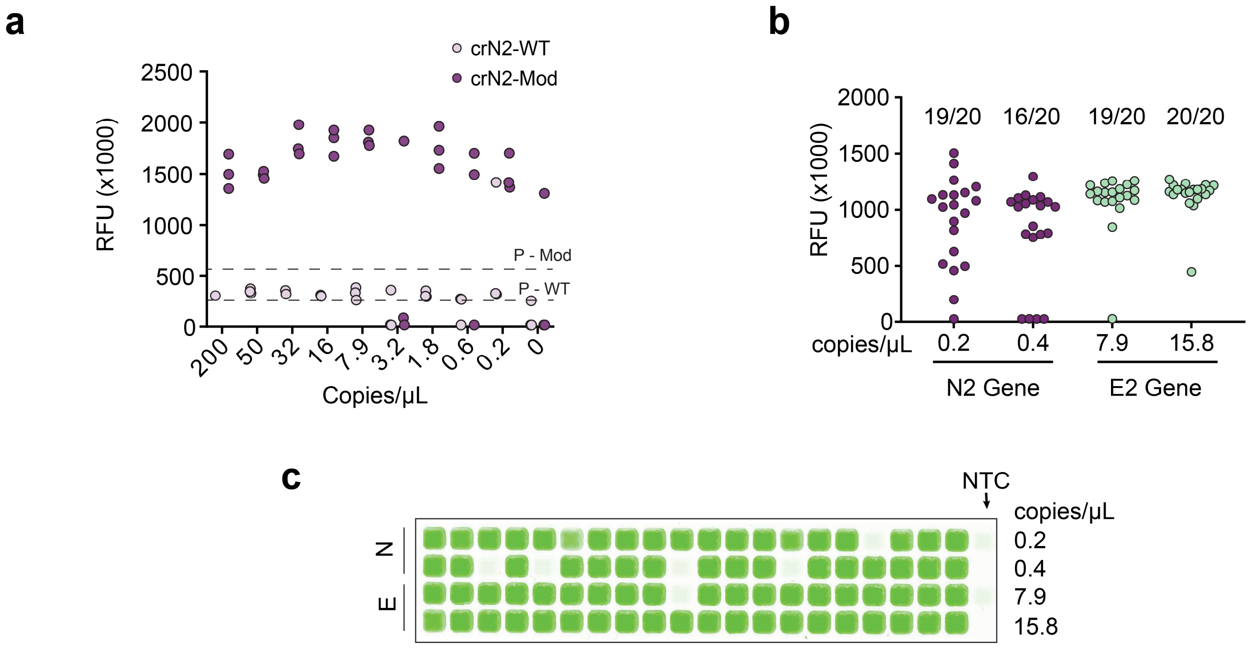

Supplementary Figure S1. LoD determination of N2 gene when UDG is not incorporated in the pre-amplification step. (**a**) Estimated LoD experiments were performed as described in figure 2 in the main text. An estimated LoD was determined based on the lowest copies/µL with 2/3 replicates detected positive. (**b**) 20 replicates with the copies/µL at 1X and 2X times the estimated LoD in (a) were repeated to confirm the final LoD. (**c**) Fluorescence image of samples in (b).

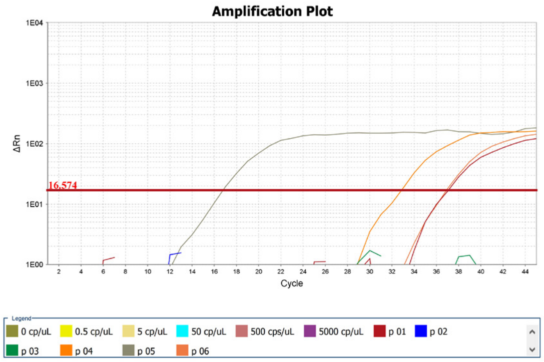

N1 Gene

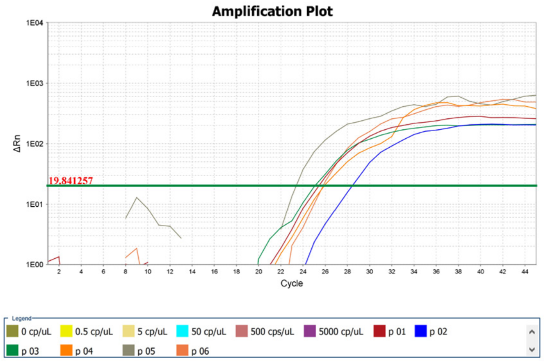

RNase P Gene

Supplementary Figure S2. Representation of clinical validation of SARS-CoV-2 detection in 62 patient samples using RT-qPCR. Obtained patient samples were re-tested with RT-qPCR following CDC-recommended protocol to determine the Ct values.

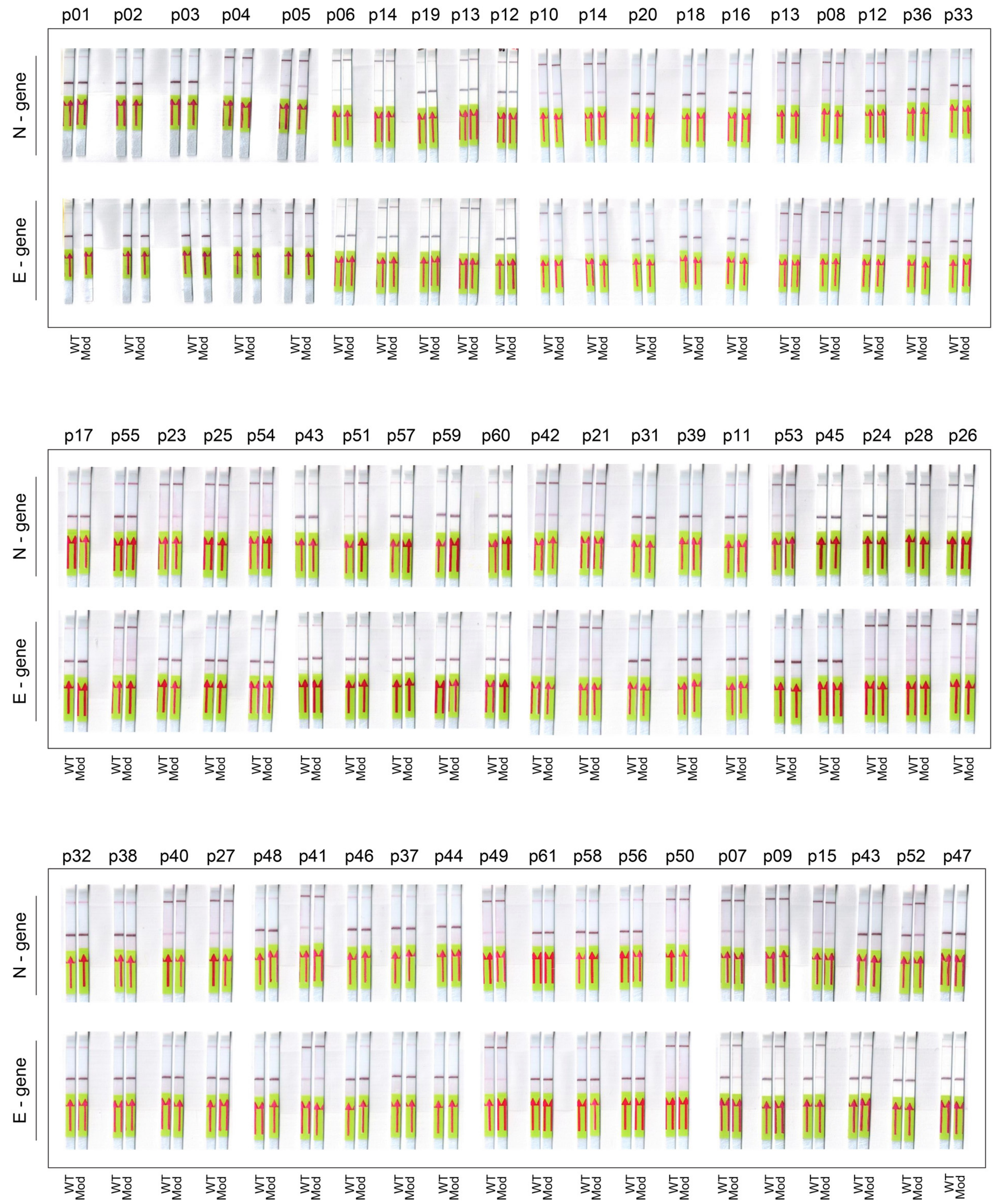

Supplementary Figure S3. Clinical validation of ENHANCE for the detection of SARS-CoV-2 in 62 patient samples using lateral flow assay. The patient samples were not in numerical order due to blind testing. The asterisk (*) denotes that patient sample 12 was repeated.

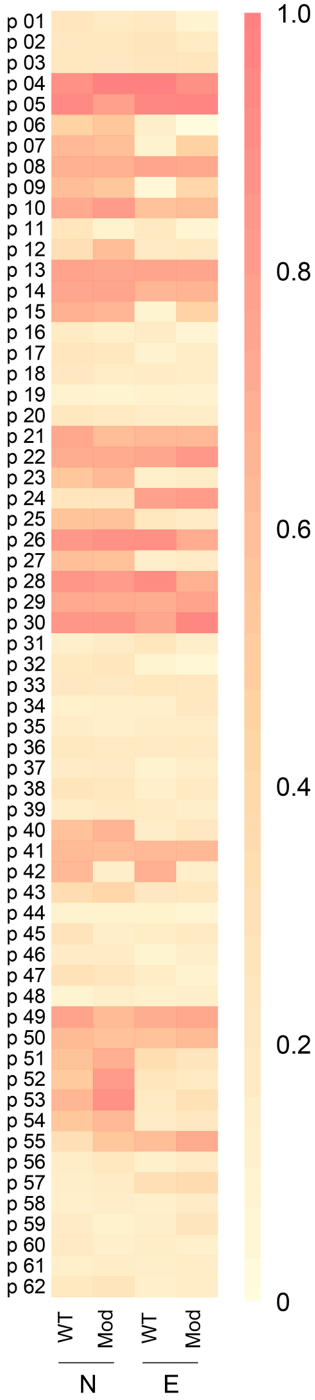

Supplementary Figure S4. Quantitative analysis of lateral flow assay using ENHANCE on 62 patient samples. The heat map shows ImageJ quantification of band intensity ratio of positive line (top band) to highest signal obtained in all 31 positive patient samples.

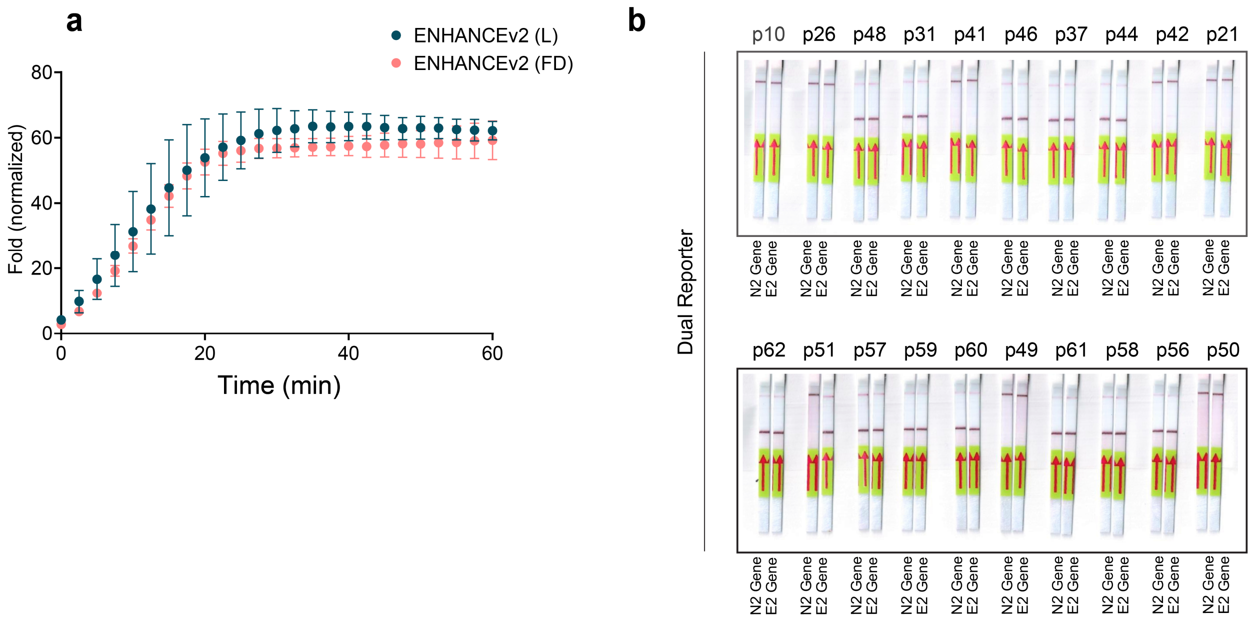

Supplementary Figure S5. Comparison between liquid version and lyophilized version of ENHANCEv2. (a) Fold change in fluorescence intensity normalized to corresponding NTC reactions targeting N2gene between liquid ENHANCEv2 (L) and freeze-dried ENHANCEv2 (FD) in one hour. Error bars represent standard deviation. (**b**) Representation of lateral flow paper strips showing compatibility of the dual reporter. Sample reactions from the fluorescence-based reporter assay using dual reporter version 2 in ENHANCEv2 was diluted down to a final concentration of 125 nM reporter followed by the lateral flow assay.

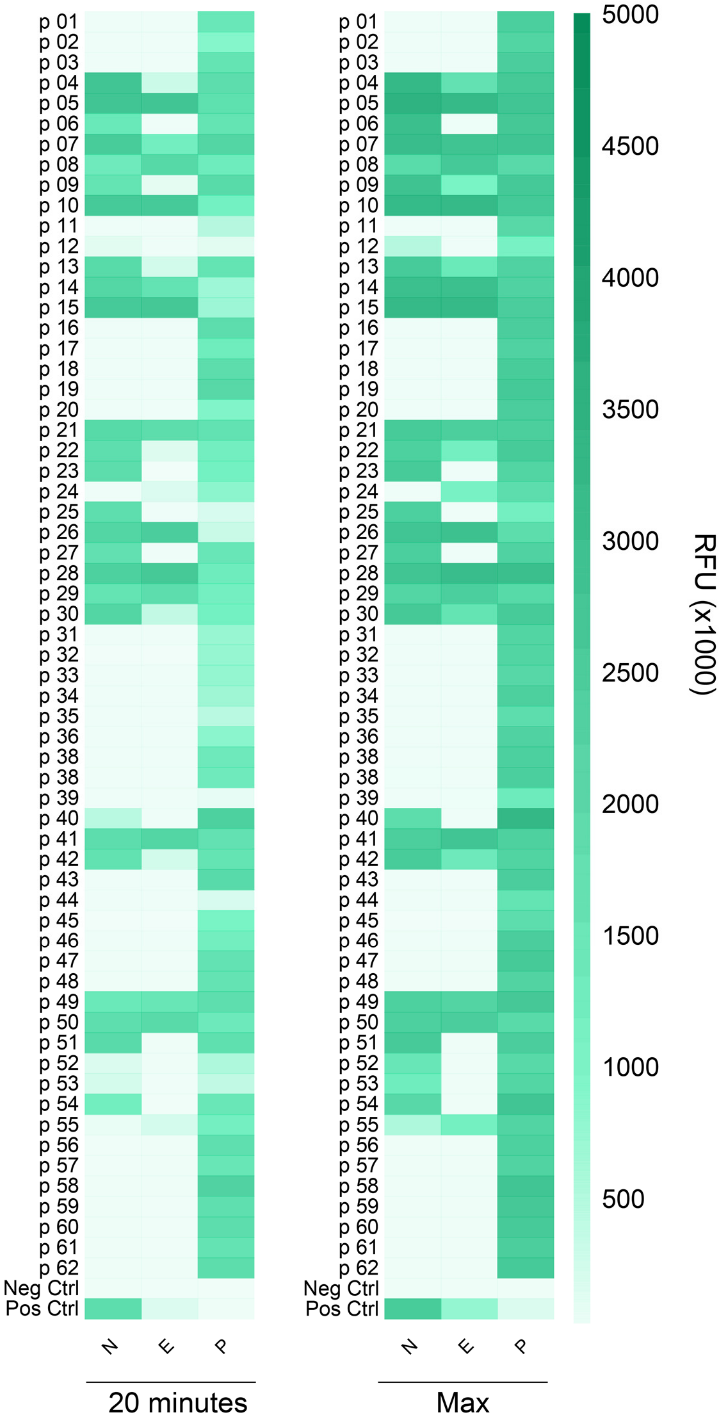

Supplementary Figure S6. Clinical validation of ENHANCEv2 for the detection of SARS-CoV-2 in 62 patient samples using fluorescence-based reporter assay. The heat map shows fluorescence intensities taken at t = 20 minutes (left) and maximum fluorescence intensities within an hour. The heat map is supplemental to fig. 4e in the main text whose fluorescence intensities were taken at t = 2.5 minutes.

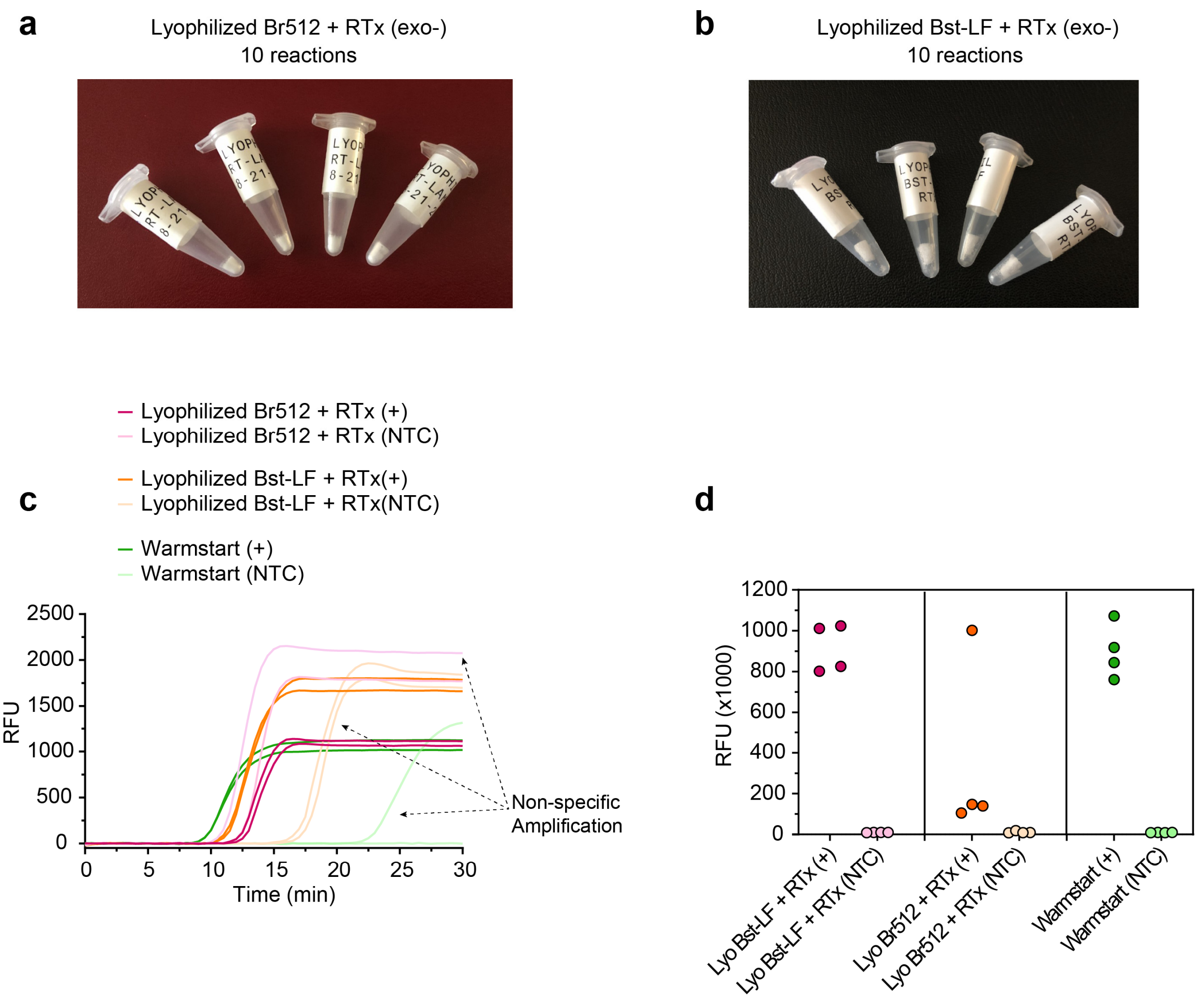

Supplementary Figure S7. Lyophilization and functional testing of RT-LAMP reactions. (a) Representation of lyophilized RT-LAMP reactions using Br512 polymerase and reverse transcriptase RTx(exo). (**b**) Representation of lyophilized RT-LAMP reactions using Bst-LF polymerase and reverse transcriptase RTx(exo). (**c**) RT-LAMP amplification of lyophilized reagents compared to commercial Warmstart® Master mix (New England Biolabs). SYTO9 dye was used to track amplification for each replicate (n = 2 biological replicates). As seen above, Bst-LF + RTx performs as robustly as the Warmstart® Master mix, whereas the Br512 + RTx combination produces non-specific signal the earliest. (+) signifies samples containing positive SARS-CoV-2. NTC denotes non-template control. (**d**) CRISPR detection reaction of amplified target using the lyophilized RT-LAMP reagents from (a) and (b) compared to the commercial Warmstart® Master mix (New England Biolabs). The fluorescence intensities were taken at t = 30 minutes, with n = 4 biological replicates.

Supplementary Table S1. Comparison of CRISPR-based detection methods

Supplementary Table S1.1 Comparing selected CRISPR-based detection methods for COVID-19

	DETECTR	SHERLOCK	StopCOVID.v2	RT-AIOD	ENHANCE
Target genes	N, E	N, ORF1ab	N	N	N, E
Enzyme	Cas12a	Cas13a	Cas12b	Cas12a	Cas12a
LoD copies/μL<br><br>(Total copies)	10-100 copies/μL<br><br>(20 copies input)	0.9-6.75 copies/μL<br><br>(7-54 copies input)	0.033 copies/μL<br><br>(100 copies per sample)	4.6 copies/μL<br><br>(5 copies input)	15-25 copies/μL<br><br>(1-40 copies input)
Time to detect patient samples<br><br>(RNA to detection)	30-40 min	60 min	45-80 min	40 min	33 min
Positive Prediction Rate	95%	96.30%	98.40%	100%	96.7%
Negative Prediction rate	100%	100%	93.40%	100%	96.7%

Supplementary Table S1.2 Benchmarking ENHANCEv2 against ENHANCEv1 & DETECTR

	DETECTR	ENHANCEv1	ENHANCEv2
Reference	Chen et al., *Science*, 2018	Nguyen et al., *Nat. Comms.*, 2020; *Methods*, 2021	This manuscript [includes ENHANCEv1 clinical validation]
Highlights	First study on Cas12a-mediated trans-cleavage activity. Several applications including COVID-19 diagnostics reported.	First study on enhancing collateral activity of Cas12a by engineering crRNAs and detecting RNA as a heteroduplex.	First study on developing and clinically validating a CRISR-based diagnostic assay with dual mode reporters. Lyophilized kit stable for at least 30 days at room temperature.
crRNA	Wild-type	Engineered: 7-nt DNA on 3' end of crRNA	Engineered: 7-nt DNA on 3' end of crRNA
Cas	LbCas12a	LbCas12a	LbCas12a^{D156R}
Kcat/Km (dsDNA)	1.7×10^7 s^{-1} M^{-1}	5.1×10^7 s^{-1} M^{-1} (3.2-fold higher)	---
Pre-amplification	DNA: RPA	DNA: RPA; RNA: RT-RPA & RT-LAMP	RNA: RT-LAMP
Targets	HPV16 & HPV18	HIV, HCV, PCA3, & SARS-CoV-2	SARS-CoV-2
Clinical validation	HPV16: 100% accuracy; HPV18: 92% accuracy	None	SARS-CoV-2: 97% accuracy
LOD range	-pre-amplification: pM; +pre-amplification: aM	-pre-amplification: fM; +pre-amplification: aM	-UDG: 0.2-7.9 copies/µL; +UDG: 15-25 copies/µL
Assay time, temperature	60-120 min, 37°C (RPA+CRISPR)	30 min, 65°C (RT-LAMP) + 10-20 min, 37°C (CRISPR)	30 min, 65°C (RT-LAMP) + 3 min, 37°C (CRISPR)
Readout mode	Single-mode: Fluorescence (FL)	Single-mode: FL or lateral flow (LF)	Dual-mode: FL+LF in one reaction
Key equipment	FL mode: Heater & plate reader	FL mode: Heater & plate reader; LF mode: Heater	FL mode: Heater & 460 nm LED; LF mode: Heater
Sample type	DNA & anal swabs	Simulated urine, DNA, RNA & heteroduplex	RNA & nasal swabs
Extraction	Crude DNA extraction	Purified DNA and RNA	Optimization: DNA extraction buffer, 15 min @ 65°C + 2 min @ 98°C; Clinical: Maxwell® RSC
Cold chain	Freezer required	Freezer required	All components lyophilized. Stable for at least 30 days at 25°C.
Cost/rxn	<$1 (F)	<$1 (F), <$3 (LFA)	<$1 (F), <$3 (LFA)

Supplementary Table S2. Assay controls and interpretation of results

Supplementary Table S2.1 Fluorescence-based reporter detection assay: Assay controls

	Positive Control	Negative Control	Interpretation
SARS-CoV-2 N2 Gene ($N2_{t=20}/N2_{t=0}$)	≥ 5	< 5	Valid for N2 gene.
	< 5	< 5	Invalid for positive control. Indicates QC failure.
	≥ 5	≥ 5	Invalid for negative control. Indicates N2 gene contamination
SARS-CoV-2 E2 Gene ($E2_{t=20}/E2_{t=0}$)	≥ 5	< 5	Valid for E2 gene.
	< 5	< 5	Invalid for positive control. Indicates QC failure.
	≥ 5	≥ 5	Invalid for negative control. Indicates E2 gene contamination
Human RNASE-P Gene ($RNASE\text{-}P_{t=20}$ / $RNASE\text{-}P_{t=0}$)	N/A	< 5	Valid for RNASE-P.
		≥ 5	Invalid. Indicates RNASE-P gene contamination.

Supplementary Table S2.2 Fluorescence-based reporter detection assay: Fold-change interpretation

SARS-CoV-2 N2 gene ($N2_{t=20}$ /$NTC_{t=20}$)	SARS-CoV-2 E2 gene ($E2_{t=20}$ /$NTC_{t=20}$)	Human RNase-P gene ($RNase\text{-}P_{t=20}$ /$NTC_{t=20}$)	Interpretation
≥ 5	≥ 5	N/A	Positive for SARS-CoV-2
≥ 5	< 5	N/A	
< 5	≥ 5	N/A	
< 5	< 5	≥ 5	Negative for SARS-CoV-2

Supplementary Table S2.3 Lateral flow assay: Assay controls

	Positive control	Negative control	Interpretation
SARS-CoV-2 N2 gene visual readout	+	-	Valid for N2 gene.
	-	+	Invalid for positive control. Indicates QC failure.
	+	+	Invalid for negative control. Indicates N2 gene contamination
SARS-CoV-2 E2 gene visual readout	+	-	Valid for E2 gene.
	-	+	Invalid for positive control. Indicates QC failure.
	+	+	Invalid for negative control. Indicates E2 gene contamination
Human RNASE-P gene visual readout	N/A	-	Valid for RNASE-P.
		+	Invalid. Indicates RNASE-P gene contamination.

Supplementary Table S2.4 Lateral flow assay: Visual readout interpretation

SARS-CoV-2 N2 gene visual readout	SARS-CoV-2 E2 gene visual readout	Human RNase-P gene visual readout	Interpretation
+	+	N/A	Positive for SARS-CoV-2
+	-	N/A	
-	+	N/A	
-	-	+	Negative for SARS-CoV-2

Supplementary References:

1. Chen, J. S. *et al.* CRISPR-Cas12a target binding unleashes indiscriminate single-stranded DNase activity. *Science* **360**, 436-+, doi:10.1126/science.aar6245 (2018).
2. Nguyen, L. T., Smith, B. M. & Jain, P. K. Enhancement of trans-cleavage activity of Cas12a with engineered crRNA enables amplified nucleic acid detection. *Nat Commun* **11**, doi:ARTN 4906.
3. Nguyen, L. T. *et al.* CRISPR-ENHANCE: An enhanced nucleic acid detection platform using Cas12a. *Methods*, doi:10.1016/j.ymeth.2021.02.001 (2021)
