# Supplementary material for: Clinical validation of engineered CRISPR/Cas12a for rapid SARS-CoV-2 detection
Source: Commun Med (Lond). 2022 Jan 12;2:7. doi: 10.1038/s43856-021-00066-4 (PMC9053293; doi:10.1038/s43856-021-00066-4)
Supplement: Supplementary file 5 — Description of Additional Supplementary Files [file 43856_2021_66_MOESM5_ESM.pdf]

Description of Additional Supplementary Files

File name: Supplementary Data 1.
Description: Source data for the main figures in 446 this manuscript.

File name: Supplementary Data 2.
Description: Oligonucleotide sequences used in the study.
