# Supplementary material for: Clinical validation of engineered CRISPR/Cas12a for rapid SARS-CoV-2 detection
Source: Commun Med (Lond). 2022 Jan 12;2:7. doi: 10.1038/s43856-021-00066-4 (PMC9053293; doi:10.1038/s43856-021-00066-4)
Supplement: Supplementary file 6 — Reporting Summary [file 43856_2021_66_MOESM6_ESM.pdf]

nature research

Corresponding author(s): Piyush K. Jain, PhD

Last updated by author(s): Nov 29, 2021

Reporting Summary

Nature Research wishes to improve the reproducibility of the work that we publish. This form provides structure for consistency and transparency in reporting. For further information on Nature Research policies, see our Editorial Policies and the Editorial Policy Checklist.

Statistics

For all statistical analyses, confirm that the following items are present in the figure legend, table legend, main text, or Methods section.

n/a	Confirmed	
☐	☒	The exact sample size (*n*) for each experimental group/condition, given as a discrete number and unit of measurement
☐	☒	A statement on whether measurements were taken from distinct samples or whether the same sample was measured repeatedly
☒	☐	The statistical test(s) used AND whether they are one- or two-sided<br>*Only common tests should be described solely by name; describe more complex techniques in the Methods section.*
☒	☐	A description of all covariates tested
☒	☐	A description of any assumptions or corrections, such as tests of normality and adjustment for multiple comparisons
☐	☒	A full description of the statistical parameters including central tendency (e.g. means) or other basic estimates (e.g. regression coefficient) AND variation (e.g. standard deviation) or associated estimates of uncertainty (e.g. confidence intervals)
☒	☐	For null hypothesis testing, the test statistic (e.g. *F*, *t*, *r*) with confidence intervals, effect sizes, degrees of freedom and *P* value noted<br>*Give P values as exact values whenever suitable.*
☒	☐	For Bayesian analysis, information on the choice of priors and Markov chain Monte Carlo settings
☒	☐	For hierarchical and complex designs, identification of the appropriate level for tests and full reporting of outcomes
☒	☐	Estimates of effect sizes (e.g. Cohen's *d*, Pearson's *r*), indicating how they were calculated

Our web collection on statistics for biologists contains articles on many of the points above.

Software and code

Policy information about availability of computer code

Data collection	No softwares were used for data collections. However, the LAMP primers were designed for each gene using the freely available PrimerExplorer software (https://primerexplorer.jp/e/). Primer sequences and crRNAs were designed and/or confirmed with nucleotide alignment for designing crRNAs and primers was performed using the Basic Local Alignment Search Tool (BLAST) tool from the National Center for Biotechnology Information (NCBI).
Data analysis	Lateral flow band intensities were later quantified by ImageJ.

For manuscripts utilizing custom algorithms or software that are central to the research but not yet described in published literature, software must be made available to editors and reviewers. We strongly encourage code deposition in a community repository (e.g. GitHub). See the Nature Research guidelines for submitting code & software for further information.

Data

Policy information about availability of data

All manuscripts must include a data availability statement. This statement should provide the following information, where applicable:

- Accession codes, unique identifiers, or web links for publicly available datasets
- A list of figures that have associated raw data
- A description of any restrictions on data availability

All data associated with this study are in the main text and the Supplementary Materials. All the raw data relevant to the manuscript is included in the source file. Additional information is available from the corresponding author upon request.

Field-specific reporting

Please select the one below that is the best fit for your research. If you are not sure, read the appropriate sections before making your selection.

☒ Life sciences ☐ Behavioural & social sciences ☐ Ecological, evolutionary & environmental sciences

For a reference copy of the document with all sections, see nature.com/documents/nr-reporting-summary-flat.pdf

Life sciences study design

All studies must disclose on these points even when the disclosure is negative.

Sample size	Sample sizes were estimated based on publications as well as recent FDA EUA approvals. The total patient samples included 62 nasal swab samples consisting of 31 predetermined SARS-CoV-2 positive samples and 31 predetermined SARS-CoV-2 negative samples.
Data exclusions	No data was excluded from this study.
Replication	Optimization of modified crRNAs for the ENHANCE platform included 20 replicates used for the limit of detection. All other replicates are repeats are indicated in the figures and/or figure captions. Evaluation of the 62 samples with ENHANCEv1 and ENHANCEv2 did not include multiple replicates per patient but rather mimics the real world testing scenario.
Randomization	Nasal swab samples were randomly selected from a larger pool of samples prior to viral RNA extraction.
Blinding	Researchers were blinded to the predetermined results of the samples prior to viral RNA extraction.

Reporting for specific materials, systems and methods

We require information from authors about some types of materials, experimental systems and methods used in many studies. Here, indicate whether each material, system or method listed is relevant to your study. If you are not sure if a list item applies to your research, read the appropriate section before selecting a response.

Materials & experimental systems

n/a	Involved in the study	
☒	☐	Antibodies
☒	☐	Eukaryotic cell lines
☒	☐	Palaeontology and archaeology
☒	☐	Animals and other organisms
☐	☒	Human research participants
☒	☐	Clinical data
☒	☐	Dual use research of concern

Methods

n/a	Involved in the study	
☒	☐	ChIP-seq
☒	☐	Flow cytometry
☒	☐	MRI-based neuroimaging

Human research participants

Policy information about studies involving human research participants

Population characteristics	Inclusion criteria:<br>1. Males/females greater than or equal to age of 2 years.<br>2. Tested positive/negative for SARS-CoV-2 by CDC recommended methods.<br>3. Collected within 10 days of onset of COVID-19 symptoms.<br><br>Exclusion criteria:<br>1. Had COVID-19 symptoms but tested negative for SARS-CoV-2.<br>2. Tested positive for SARS-CoV-2 but the samples were collected >=11 days post-onset of symptoms.
Recruitment	Only de-identified human samples were obtained from the University of Florida (UF) Clinical and Translational Science Institute (CTSI) Biorepository and the commercial vendor, Boca Biolistics, by following the guidelines listed in the UF Institutional Review Board (IRB) protocol IRB202000781. Due to limited availability of the clinical specimens during our study, the first available samples that met the population characteristics were obtained to avoid any bias. Only de-identified samples were collected without any identifiable information or informed consent.
Ethics oversight	This study was performed under the UF IRB protocol IRB202000781, which was approved as a non-human study, and all relevant ethical regulations were followed. De-identified human samples were obtained from the UF CTSI Biorepository, collected under the UF IRB approved protocol IRB20200879, and from the Boca Biolistics, procured under the IIRB delinking protocol SOP 10-00114 Rev E.

The CTSI Biorepository was approved to collect specimens without informed consent due to the COVID19 pandemic being an unprecedented public health emergency and it would limit the research if all samples were not included. There was also the option of obtaining informed consent wherever possible. There were specific limits in the amount and type of data allowed to be gathered for those samples collected without informed consent. Some samples being tested for COVID 19 by the UF Pathology Lab will come from patients in outlying clinics or hospitals. Although PHI was collected with the samples, no identifiable data or tissue was nor will be subsequently dispensed. All connections of tissue with data have been and will be conducted by honest brokers.

Boca Biolistics is an FDA-recommended provider of SARS-CoV-2 biospecimens for research and diagnostic development. BBL provides remnant SARS-CoV-2 swab specimens as remnant (leftover) samples procured from our network of CAP/CLIA accredited partner laboratories across the United States all of whom have been instrumental in providing COVID-19 screening throughout the pandemic. Under Boca's IIRB Delinking protocol samples are procured and de-linked so that no information can be traced back to the individual patient, providing sound and secure de-identification protecting patient identity. Boca's SOP is consistent with the FDA's "Guidance on informed consent for in vitro diagnostic device studies using leftover human specimens that are not individually identifiable". This allows BBL to provide tens of thousands of highly needed SARS-CoV-2 swab specimens that have been instrumental in both the development and validation of diagnostic instruments throughout the world to test for COVID-19.

Note that full information on the approval of the study protocol must also be provided in the manuscript.
